# A High-Throughput Single-Clone Phage Fluorescence Microwell Immunoassay and Laser-Driven Clonal Retrieval System

**DOI:** 10.3390/biom10040517

**Published:** 2020-03-29

**Authors:** Seohee Chang, Soohyun Kim, Jerome Han, Suji Ha, Hyunho Lee, Seo Woo Song, Daewon Lee, Sunghoon Kwon, Junho Chung, Junhoi Kim

**Affiliations:** 1 Department of Electrical and Computer Engineering, Seoul National University, Seoul 08826, Korea; seohee0708@snu.ac.kr (S.C.); eyebis208@gmail.com (H.L.); skwon@snu.ac.kr (S.K.); 2 Department of Biochemistry and Molecular Biology, Seoul National University College of Medicine, Seoul National University, Seoul 03080, Korea; kimchii481@gmail.com (S.K.); wsukh23@gmail.com (J.H.); tnwlgk123@gmail.com (S.H.); 3 Cancer Research Institute, Seoul National University College of Medicine, Seoul National University, Seoul 03080, Korea; 4 Department of Biomedical Science, Seoul National University College of Medicine, Seoul National University, Seoul 03080, Korea; 5 Bio-Max Institute, Seoul National University, Seoul 08826, Korea; ssw0313@gmail.com; 6 BK21+ Creative Research Engineer Development for IT, Seoul National University, Seoul 08826, Korea; leex7700@snu.ac.kr

**Keywords:** phage display, single clone, microchip

## Abstract

Phage display is one of the most frequently used platform technologies utilized to screen and select therapeutic antibodies, and has contributed to the development of more than 10 therapeutic antibodies used in the clinic. Despite advantages like efficiency and low cost, it has intrinsic technical limitations, such as the asymmetrical amplification of the library after each round of biopanning, which is regarded as a reason for it yielding a very limited number of antigen binders. In this study, we developed a high-throughput single-clonal screening system comprised of fluorescence immunoassays and a laser-driven clonal DNA retrieval system using microchip technology. Using this system, from a single-chain variable fragment (scFv) library displayed on phages with a complexity of 5.21 × 10^5^ harboring random mutations at five amino acid residues, more than 70,000 clones—corresponding to ~14% of the library complexity—were screened, resulting in 78 antigen-reactive scFv sequences with mutations restricted to the randomized residues. Our results demonstrate that this system can significantly reduce the number of biopanning rounds, or even eliminate the need for this process for libraries with lower complexity, providing an opportunity to obtain more diverse clones from the library.

## 1. Introduction

Phage display is a widely used technology for screening antibody libraries in therapeutic drug discovery [1,2,3,4,5,6,7,8]. A variety of antibodies encoded in the form of a phagemid library can be displayed on the surface of phages, providing genotype-to-phenotype linkage and making them useful for antibody selection and identification [9,10]. The biopanning process selectively enriches a set of antibodies with affinity to a given antigen so that a series of binders can be selected out of a large number of non-binding clones. This tremendous screening capability, along with several previously reported advantages [11,12,13], has made phage display the gold standard for antibody drug discovery.

Despite these benefits, phage display technology has intrinsic technical limitations. Laborious colony picking and the functional testing of individual phage clones restrict the practical screening size to the thousands, which is a fraction of the required level [14]. Even with the help of automated instruments such as colony pickers [15,16,17], the gap between practical and ideal screening capacities cannot be bridged. This necessitates multiple rounds of biopanning, which typically takes weeks. Loss of the best binders is also a critical problem inherent in phage display. Rare binders can be lost due to the strong amplification bias introduced during successive biopanning processes [18,19]. Furthermore, insufficient elution can cause the loss of strong binders, excluding them from the subsequent steps [20]. The development of an elaborate approach addressing these technical issues is required to minimize labor-intensive work, save considerable time, and reduce the possibility of losing the best binders in the process.

Considerable effort has been made to adopt microtechnologies which advance the field of bioscience [21,22,23]. For phage display applications, various single-cell-level analyses were developed to minimize amplification bias and maximize throughput, thereby reducing the number of biopanning rounds. By employing a microcapillary device with densely packed microreaction chambers, more than one million antibody clones were successfully screened at once [24]. A microfluidic device was also utilized to generate water-in-oil microemulsion reaction chambers, theoretically increasing the analysis throughput to infinite [25,26]. Although both methods could reduce the number of required biopanning rounds based on their extremely high throughput, they require complicated sample isolation approaches. Furthermore, both methods are vulnerable to possible sample cross-contamination during sample retrieval [27,28,29].

In this study, we describe a high-throughput single-clonal screening system comprised of fluorescence immunoassays as well as a laser-driven clonal DNA retrieval system using microchip technology. The use of a single-clone-level approach in combination with an elaborate sample retrieval method enabled high-throughput sample retrieval with minimal amplification bias and sample cross-contamination.

The efficiency of this system was tested by using a single-chain variable fragment (scFv) library displayed on phages with a complexity of 5.21 × 10^5^, harboring random mutations at five amino acid residues. Without biopanning, we could screen 78 antigen-reactive (AR) scFv sequences with mutations, restricted to the randomized residues when 70,000 clones were screened in parallel. We believe that the result is superior, or at least equivalent, to the conventional biopanning and screening procedure.

## 2. Materials and Methods

### 2.1. Microwell Array Chip Fabrication

The microwell array chip was prepared by assembling a polymeric microwell array and a sample-capturing substrate. The microwell array was fabricated using soft lithography, in which polydimethylsiloxane (PDMS, SYLGARD 184, Dow, Midland, MI, USA) was cast onto a silicon mold with an SU-8 photoresist pattern (SU-8 2015, Microchem, Westborough, MA, USA) and cured at 95 °C for 1 h [30,31]. The cured 3-mm-thick PDMS block, with a two-dimensional array of cylindrical microwells (d ~ 60 µm, h ~ 40 µm), was then peeled off from the mold. The sample-capturing substrate was prepared by forming a sample-capturing layer on top of an indium tin oxide (ITO)-coated glass slide (ITO thickness ~100 nm, Fine Chemicals, Seoul, Korea). For the sample-capturing layer formation, a mixture of 3.4 mL of (7.0–8.0% vinylmethylsiloxane)–dimethylsiloxane copolymer (VDT-731); 18 µL of platinum–divinyltetramethyldisiloxane (SIP6831.2LC); 1 mL of (25–35% methylhydrosiloxane)–dimethylsiloxane copolymer (HMS-301); and 100 µL of 1,3,5,7-tetravinyl–1,3,5,7-tetramethylcyclotetrasiloxane (SIT7900.0, all from Gelest, Morrisville, PA, USA) was spin-coated and thermally cured [32,33,34], yielding a polymeric layer with a thickness of 7 µm. The microwell array and sample-capturing substrate were directed towards each other and fixed using a custom-made chip holder consisting of an aluminum plate and acryl sheet. The chip holder was designed to fit into a conventional microplate reader for optical imaging through a transparent window, while maintaining reliable compartmentalization of individual microreaction chambers with tightening screws.

### 2.2. Library Preparation

Three anti-human hepatocyte growth factor (HGF) scFvs and anti-cotinine scFv (three AR phage clones (P1, P2, and P3) as well as an antigen non-reactive (NR) phage clone) were selected from a previous study [35] for validation experiments. Each scFv gene was inserted into a pComb3XSS phagemid vector, and phages encoding AR P1, P2, P3, or NR were mixed in various ratios for use as alternatives for the phage library to be screened against human HGF. For deimmunization screening, a pre-discovered anti-CD28 scFv clone was selected as a randomization template in which five amino acid residues were mutated (RMN/YMY/NNK/MTN/RYN), yielding a synthetic library with a complexity of 5.21 × 10^5^. A set of degenerate Ultramer DNA oligonucleotides (Integrated DNA Technologies, Coralville, IA, USA) was used to construct the randomization library as described previously [36]. In all experiments, phages were rescued before use, as previously reported [37].

### 2.3. Phage Display Preparation

The microwell array and the sample-capturing substrate were treated with an oxygen plasma (CUTE, Femto Science, Hwaseong, Korea) for 2 min. The plasma-treated pair was then immersed in an antigen solution (5 µg/mL in 0.1 M sodium bicarbonate buffer, pH 8.6) at 37 °C for 90 min, followed by blocking with 3% bovine serum albumin (BSA) in phosphate-buffered saline (PBS) at room temperature for 1 h. *Escherichia coli* (*E. coli*) cells were grown in 300 µL of super broth (SB) medium at 250 rpm/37 °C until reaching an optical density at 600 nm of ~0.7, followed by phage infection (15 min, room temperature) with a 100:1 cell-to-phage ratio. *E. coli* cells were then incubated at 250 rpm/37 °C for 2 h after a two-step addition of carbenicillin (final concentration ~40 µg/mL; Sigma-Aldrich, St. Louis, MO, USA). Finally, the *E. coli* cell suspension concentration was adjusted to a single-cell loading condition with SB medium containing helper phages (final concentration ~10^11^ pfu/mL; M13K07, New England Biolabs, Ipswich, MA, USA) and carbenicillin (final concentration ~50 µg/mL). The prepared *E. coli* solution was immediately loaded into the microwell array.

### 2.4. On-Chip Phage Display Experiment

Immediately after *E. coli* solution loading, the microwell array was mounted onto the chip holder with the sample-capturing substrate in order to separate individual microwells. The chip-loaded holder was incubated at 37 °C overnight in order to allow cell growth and phage production within microwells. The microwell array chip was disassembled back to the microwell array and sample-capturing substrate, both of which were washed with 0.05% Tween 20 in PBS (PBST). The microwell array was immersed in 3% BSA in PBS at room temperature for 1 h, followed by incubation with fluorescein isothiocyanate (FITC)-conjugated anti-M13 antibody (61R-M101AFT, Fitzgerald, Acton, MA, USA) for phage fluorescence microwell immunoassays. The labeled microwell array was washed with PBST, assembled on a non-treated glass slide, and mounted on the chip holder for subsequent imaging. The sample-capturing substrate was also treated with the same blocking and labeling conditions in validation experiments.

### 2.5. Image Acquisition

Brightfield and fluorescence images of the microwell array chip were acquired using an automated fluorescence microscope (Ti-E, Nikon, Tokyo, Japan) equipped with a high-sensitivity charge-coupled device camera (C11440, Hamamatsu Photonics, Hamamatsu, Japan). For fluorescence imaging under the 470 nm excitation condition, a light-emitting diode illuminator (Spectra X 6-NII-SA, Lumencor, Beaverton, OR, USA) was used in combination with an FITC filter unit (excitation peak ~490 nm, emission peak ~525 nm). Across all imaging conditions, a whole microwell area within the microwell array chip was scanned with a 4× objective lens (CFI Plan Fluor 4×, Nikon), generating a series of microscope images with a field-of-view of 3328 µm × 3328 µm. The image acquisition process was performed both before and after microwell array chip disassembly.

### 2.6. Image Analysis

Individual images were stitched into a large reconstructed image showing the entire microwell array chip [38]. A Python script for image analysis was developed and utilized in order to identify the positions of target samples to be retrieved from the chip. More specifically, coordinates of individual microwells and their corresponding fluorescence intensities were first extracted from the stitched images. By comparing the coordinates of the microwells and on-chip alignment markers obtained before and after chip disassembly, target sample positions were then determined on the sample-capturing substrate. For coordinate transformation, a simple first-order equation was employed to precisely compensate for distortion of the elastomeric device caused during chip disassembly. The obtained positions of the target samples were finally converted into the displacements needed for the operation of a laser-driven sample retrieval system.

### 2.7. Target Sample Retrieval and Sequencing

The previously developed sample retrieval system [39,40] was utilized under the control of the Python script written for this work. To summarize, two independent 3-axis mechanical stages and an infrared pulse laser (MINILITE II, Continuum Lasers, San Jose, CA, USA) were operated in a synchronized manner to selectively retrieve phages of interest from the surface of the sample-capturing substrate. The retrieved phages were collected into an 8-strip polymerase chain reaction (PCR) tube filled with 7 µL of phage lysis buffer (1% Triton X-100, 500 mM guanidine-HCl, 10 mM MOPS, pH 6.5), followed by 20-min incubation at 80 °C in order to lyse the phage coat protein. Phage viral DNA covering the scFv gene was then amplified using Jumpstart DNA polymerase (D9307, Sigma-Aldrich) and then sequenced by the Sanger method. For high-throughput analysis of the collected samples, multiple samples were pooled into a single tube before phage lysis and converted into plasmid vector form after amplification. High-throughput clonal retrieval and sequencing were performed using TrueRepertoire technology (TR5000, Celemics, Seoul, Korea) [41].

### 2.8. Clone Identification and Validation

The sequencing results were analyzed using a Python script written for identification of scFv clones and mutations as follows: (i) Light- and heavy-chain sequences were extracted from the obtained reads by aligning to the vector and scFv linker sequence, (ii) sequences with unintended mutations were excluded, and (iii) sequences with amino acid mismatches compared to the wild-type clone were filtered out. In order to confirm that the identified clones had effective antigen reactivity, phages were rescued from the individualized colony samples and subjected to phage enzyme-linked immunosorbent assays (ELISAs) as previously described [42]. The mutation frequency pattern was obtained from the identified AR scFv clones.

## 3. Results and Discussion

### 3.1. Cloning, Phage Fluorescence Microwell Immunoassay, and Laser-Driven Retrieval of Phage Clones

**Figure 1:**
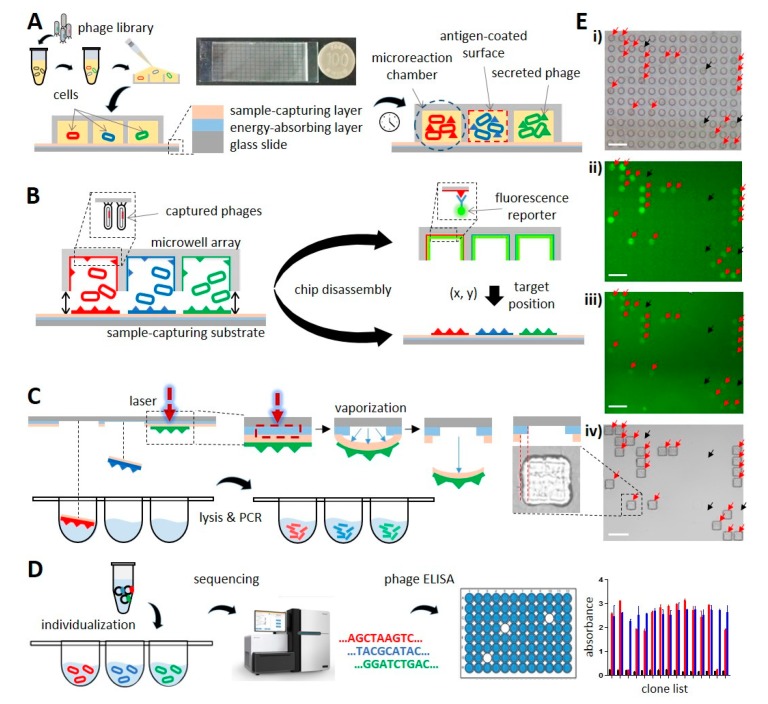
A schematic view of our platform. (**A**) Individualized growth of phage-infected cells. A microwell array and sample-capturing substrate were assembled to separate bacterial cells within microreaction chambers. (**B**) Disassembly of the microwell array chip. The microwell array was labeled with a fluorescence reporter for the phage fluorescence microwell immunoassay. (**C**) Laser-driven sample retrieval. Phages captured on a sample-capturing layer were retrieved, and their phagemid DNAs were amplified. The inset shows an image of the sample-capturing substrate after pulse-laser irradiation. (**D**) Sequencing analysis of retrieved phage clones. The selected samples were subjected to high-throughput clonal retrieval and phage ELISA. (**E**) Representative microscopy images corresponding to each step of the workflow: brightfield image of the assembled microwell array chip (**i**), fluorescence images of the microwell array (**ii**) and sample-capturing substrate (**iii**) after fluorescence reporter conjugation, and brightfield image of the sample-capturing substrate (**iv**) after sample retrieval. Cell-occupied microwells with/without fluorescence signals and the corresponding spots on the sample-capturing substrate are indicated by red/black arrows, respectively. All scale bars are 200 μm.

A key idea of this study was to perform phage cloning and single-cell level phage fluorescence microwell immunoassays with the help of microwell technology. To achieve this, individual *E. coli* cells carrying phagemid clones were separated from each other in an independent microwell environment. Then, *E. coli* cells were allowed to grow, while phages displaying scFvs encoded by the phagemid DNA were rescued. The rescued phages interacted with the antigen-coated surface of the microreaction chamber (Figure 1A). A parallel reaction performed in an array of identical microwells enabled library screening at single-cell resolution in a nearly identical environment. The microwell array chip was disassembled into two components (Figure 1B, microwell array and sample-capturing substrate), which were subjected to different experiments. The microwell array was used for phage fluorescence microwell immunoassays. The microwell array was first washed and incubated with FITC-conjugated anti-M13 antibody. Then, fluorescence images of the microwell array chip were acquired in order to obtain the locations of the AR phage clones (Figure 1E(ii)). Next, the AR phage clones were retrieved from the three-layer sample-capturing substrate. The sample-capturing layer was prepared to reliably capture the phages by spin-coating a mixture of vinylmethylsiloxane–dimethylsiloxane copolymer; platinum–divinyltetramethyldisiloxane; methylhydrosiloxane–dimethylsiloxane copolymer; and 1,3,5,7-tetravinyl–1,3,5,7-tetramethylcyclotetrasiloxane and thermal curing.

The energy-absorbing layer was made of ITO coated on a glass slide. By irradiating with an infrared laser pulse, the sample-capturing layer of the substrate at the depicted location was sheared off by the pressure generated through the vaporization of the energy-absorbing layer and collected into individual wells of the microtiter plate, as depicted in Figure 1C. After phagemid DNA was extracted from the phage, the scFv gene was amplified and subjected to a high-throughput clonal retrieval system as described previously [41]. The retrieved phage clones were used in phage ELISA in order to confirm the antigen reactivity (Figure 1D).

### 3.2. Designing the Microwell Array Chip and Establishment of the Cloning Procedure

**Figure 2:**
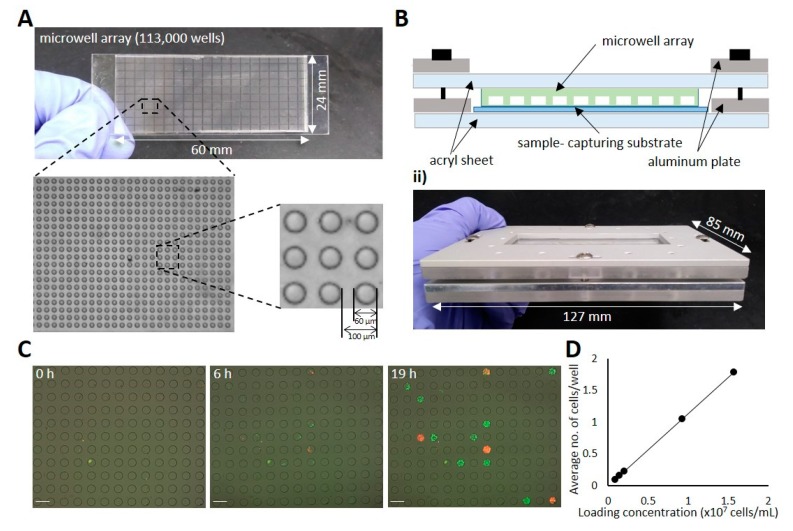
Microwell array chip. (**A**) Photograph of a microwell array with 113,000 microwells. (**B**) Microwell array chip fixed by a chip holder. (**C**) Microscopy images of microwells loaded with a mixture of red fluorescent protein (RFP)-expressing cells or green fluorescent protein (GFP)-expressing cells. Brightfield and fluorescence images were acquired after 0, 6, and 19 h of incubation and merged for visualization. All scale bars are 200 µm. (**D**) The relationship between cell loading concentration and the average number of cells trapped in microwells.

The microwell array chip was prepared to have an array of microwells in a standard glass slide format (Figure 2A). After testing different microwell dimensions, a microwell volume of 113 pL was found to allow sufficient growth and viability of loaded *E. coli* cells for at least 48 h, resulting in 113,000 microwells on a single microwell array chip. The chip holder was designed to have an acryl-aluminum hybrid structure for imaging compatibility and mechanical stability as shown in Figure 2B. The central acrylic window enables optical imaging of the chip while maintaining tight contact between the microwell array and the sample-capturing substrate with aluminum supports.

To verify the suitability of the designed microwell array chip and holder, bacterial cell growth was monitored as shown in Figure 2C. After 19 h of incubation with a mixture of red fluorescent protein (RFP)- or green fluorescent protein (GFP)-expressing bacterial cells, microwells were filled with single-colored cells. To determine optimal cell loading conditions, a simple Poisson distribution model was adopted; 95% of cell-occupied microwells contained single cells when 10% of the microwells were occupied by cells. A series of diluted cell suspensions was tested prior to all experiments to adjust the average number of cells in a microwell to 0.1 (Figure 2D), which is equivalent to more than 10,000 assays with a single chip.

### 3.3. Validation Using Phage-Displayed scFv Clones

**Figure 3:**
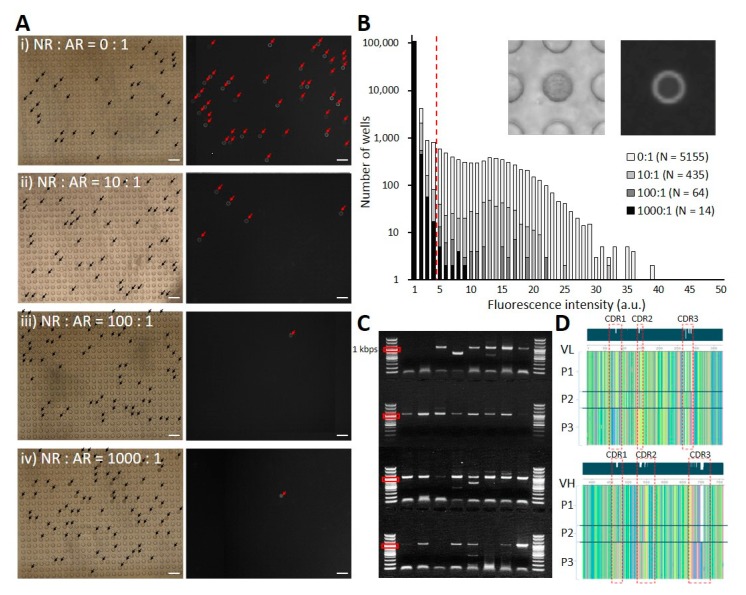
Validation experiments. (**A**) Microscopy images of microwells before (left, brightfield) and after (right, fluorescence) chip disassembly. The ratios of non-reactive (NR):antigen reactive (AR) phage clones were adjusted to 0:1 (**i**), 10:1 (**ii**), 100:1 (**iii**), and 1000:1 (**iv**), respectively. Arrows indicate microwells showing distinct cell occupancy (black) or fluorescence signal (red). All scale bars are 200 μm. (**B**) Distribution of fluorescence intensities extracted from the four experiments. The red dashed line indicates the fluorescence intensity threshold for AR signals. Inset shows microscopy images of a representative microwell. (**C**) Representative gel electrophoresis result of single-chain variable fragment (scFv) products amplified from retrieved clones. (**D**) Multiple sequence alignment result of identified clones. Three different AR P1, P2, and P3 phage clones were mixed in a ratio of 1:1:1 at the time of cell infection.

To confirm that the developed platform works as desired, a 1:1:1 mixture of three AR phage clones (P1, P2, and P3) was made (sample 1). Then sample 1 was diluted with an NR clone at three different ratios (1:10, 1:100, and 1:1000) to obtain sample 2, sample 3, and sample 4. Next, these four samples were subjected to cloning in microwell array chips. In phage fluorescence microwell immunoassays, 5155, 435, 64, and 14 microwells (samples 1, 2, 3, and 4) were found to have distinct fluorescence intensity levels in comparison to empty microwells (Figure 3A,B). For sequence verification, 48 phage clones from each sample were selected (samples 1–3, Figure 3A(i–iii)) and subjected to scFv amplification and nucleotide sequencing (Figure 3C). A subset of phage clones (15.5%) contained noisy base callings in sequencing and were excluded from further analysis. Microwells with multi-cell seeding events, which occurred in ~5% of the non-empty microwells at a cell loading concentration of 0.1 cell/well, contained mixed samples and partially accounted for the noisy base calling. The sequences of AR P1, P2, and P3 phage clones were obtained at a ~3:1:2 ratio, while the NR clone sequence was not detected (Figure 3D). The fluorescence intensity of the AR P1 phage clone was generally higher than those of P2 and P3, possibly reflecting a higher relative retrieval frequency of P1. Based on these results, we concluded that our system functioned exactly as expected.

### 3.4. Application to the Phage-Displayed scFv Library

The immunogenicity of therapeutic antibodies can drive an anti-drug immune response that compromises efficacy and undermines safety [43]. The presence of human T-cell epitopes within the antibody can activate helper T cells, neutralizing the therapeutic effect [44,45,46]. A range of strategies, including site-directed mutagenesis, have been established to delete T-cell epitopes; this strategy is then followed by high-throughput functional screening in order to test antibody immunogenicity [47,48].

**Figure 4:**
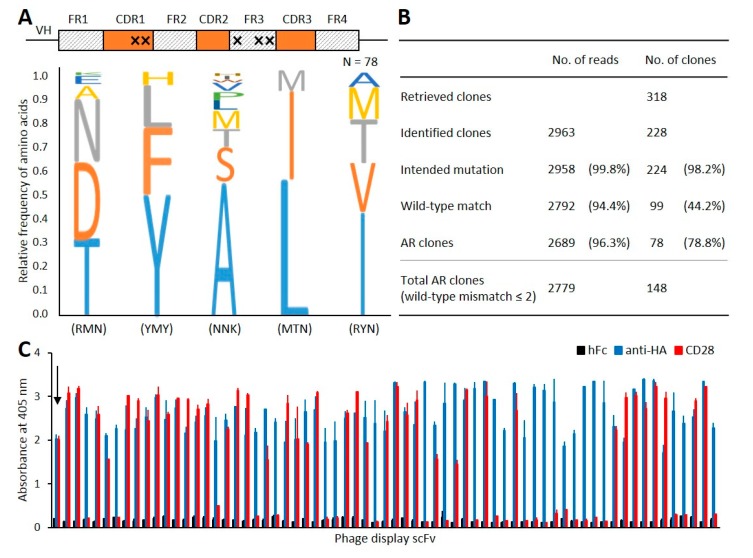
Binder identification from the phage fluorescence microwell immunoassay of a mutagenesis deimmunization library. (**A**) Mutation frequency patterns in the five randomization residues. Two residues in HCDR1 and three residues in HFR3 were randomized from the wild-type clone. (**B**) Analysis of high-throughput sequencing data for the identification of scFv clones as well as valid mutations. (**C**) Representative monoclonal phage ELISA result. The arrow indicates a wild-type clone. Each bar represents the absorbance value against hFc fusion protein (negative control, black), anti-HA antibody (positive control, blue), and CD28 fusion protein (target, red), respectively.

In this work, a mutagenesis library with random mutations at five amino acid residues in the heavy-chain variable region was synthesized for deimmunization and subjected to our platform without a preconditioning biopanning process (Figure 4A). Using seven microwell array chips, more than 70,000 phage clones were screened, corresponding to ~14% of the library diversity. In phage fluorescence microwell immunoassays, 318 microwells (AR hit ratio ~0.45%) exhibited distinct fluorescence signals, all of which were subjected to sequencing analysis. After excluding sequences with defective variable chains, codon frameshifts, or low-quality scores, we successfully identified 228 unique scFv clones (total read count ~2963). The read counts of the most frequent clone and the second most frequent clone were 1823 and 584, respectively, accounting for 81.2% of the total read count. The recovered scFv clones with unintended mutations or amino acid mismatches, compared to the wild-type clone, were further filtered out, leaving 99 clones. The antigen reactivity of clones was measured using phage ELISA. When the absorbance of the tested clones exceeded the mean plus five standard deviations of that of negative control wells (absorbance ≥ 0.4), we designated the clone as an AR clone. As a result, 78 out of 99 clones (78.8%) were finally confirmed as AR clones with the mutation frequency pattern shown in Figure 4A. The other clones were regarded as false-positive clones, generated by nonspecific binding of the overexpressed low-affinity clones to the antigens within the microwells. When the filtering condition was extended to allow up to two amino acid mismatches on unintended amino acid residues, the number of discovered AR clones increased to 148 (Figure 4B,C). Those mismatches could have originated from undesired mutational events introduced by PCR, possibly having no effect on their affinities and thereby increasing the final number of identified AR clones. Among the additional 70 AR clones obtained, 47 were indeed found to have exactly the same mutation pattern, with one of those perfectly matched to the wild-type clone.

## 4. Conclusions

We developed a system that enables the determination of the antigen reactivity of individual phage clones and clonal retrieval of AR phage clones in a very-high-throughput manner, reducing the need for biopanning for enrichment and possibly eliminating the process entirely for libraries with low complexity. Our system enables the screening of an antibody library with minimal amplification bias in order to increase the likelihood of discovering rare binders, and will be further improved to enable the screening of naïve libraries with higher complexity, without the biopanning process.
